# The effectiveness of fixed speed cameras on Iranian taxi drivers: An evaluation of the influential factors

**DOI:** 10.3389/fpubh.2022.964214

**Published:** 2022-08-30

**Authors:** Mohammad-Reza Malekpour, Sina Azadnajafabad, Sahba Rezazadeh-Khadem, Kavi Bhalla, Erfan Ghasemi, Seyed Taghai Heydari, Seyyed-Hadi Ghamari, Mohsen Abbasi-Kangevari, Nazila Rezaei, Mahmoud Manian, Saeid Shahraz, Negar Rezaei, Kamran B. Lankarani, Farshad Farzadfar

**Affiliations:** ^1^Non-Communicable Diseases Research Center, Endocrinology and Metabolism Population Sciences Institute, Tehran University of Medical Sciences, Tehran, Iran; ^2^Public Health Sciences, University of Chicago, Chicago, IL, United States; ^3^Health Policy Research Center, Institute of Health, Shiraz University of Medical Sciences, Shiraz, Iran; ^4^Faculty of Computer Engineering and Science, Shahid Beheshti University, Tehran, Iran; ^5^Institute for Clinical Research and Health Policy Studies, Tufts Medical Center, Boston, MA, United States; ^6^Endocrinology and Metabolism Research Center, Endocrinology and Metabolism Clinical Sciences Institute, Tehran University of Medical Sciences, Tehran, Iran

**Keywords:** public health, urban planning, road traffic injury, Asian cities, accident prevention, fixed speed camera

## Abstract

**Background:**

The adherence to speed limits can reduce deaths associated with road traffic injuries (RTIs) by more than a quarter. This study aimed to evaluate the effective factors on the speeding behavior of Iranian taxi drivers around fixed speed cameras.

**Method:**

Telematics devices used in this study collected the location and speed of the vehicles. The units of analysis in this study were trips per camera, including 2.5 km before and after each camera. Linear regression analysis was used to identify kangaroo driving (KD), defined as trips with a significant V-shape in speed distribution around the camera. In the clustered camera zones, cameras were placed at regular intervals of approximately 3.5 km.

**Findings:**

A total of 93,160 trips were recorded from 13,857,443 data points. There was an inverse association between drivers' age and KD with an odds ratio (OR) of 0.98 (95% confidence interval: 0.98–0.98). The intercity trips had a substantially higher probability of KD than urban trips (OR: 4.94 [4.73–5.16]). The tendency of drivers toward KD during the daylight hours vs. nighttime was significant for both urban (OR: 1.15 [1.06–1.25]) and intercity (OR: 1.18 [1.14–1.22]) trips. The 4 −8 a.m. period had the highest chance of KD in both urban (10.71% [7.41–14.53]) and intercity (44.13% [41.18–47.09]) trips. There was a significant decrease in the odds of KD inside the clustered camera zones (OR: 0.22 [0.20–0.25]).

**Conclusion:**

The heterogeneous occurrence of KD in different locations and times indicates the necessity of evidence-based decision-making in urban planning to improve safe driving behaviors. The lower occurrence of KD in clustered camera zones could be a practical key to the effective control of speeding behaviors by helping in the efficient placement of cameras through sustainable development of cities and roads.

## Introduction

Road traffic injuries (RTIs), with the contribution to 2.1% of deaths and 2.9% of disability-adjusted life years (DALYs), were the 12th and 7th leading causes of death and DALYs worldwide in 2019, respectively (1). Strikingly, according to the World Health Organization (WHO) report on the global status of road safety in 2018, RTIs were the leading causes of death in the 5–29 age group (2). In terms of transport injury rate, Iran, with 26.6 deaths per 100,000, ranked 22nd worldwide and 9th in North Africa and the Middle East in 2019 (1). In addition, transport injuries, with more than 5.7% of total deaths, was the fourth leading cause of mortality among Iranians in 2019 (1).

As a proven risk factor for RTIs, speed increases both the incidence and severity of traffic injuries (3–5). While a 5% increase in average speed could result in approximately 20% more fatal RTIs (6), adherence to speed limits can reduce RTI-associated mortality by more than a quarter (7). Nevertheless, between 40 and 50% of drivers exceed the posted speed limit (6, 8, 9). In addition, it has been shown that professional drivers often suffer from work-related stresses, such as time pressure, extended work schedules, and environmental overstimulation (10, 11). Thus, taxi drivers, as a considerable portion of the professional drivers, are at a higher risk of engaging in speeding and unsafe driving behaviors (12–16).

Among various traffic calming measures, the implementation of fixed speed cameras (FSCs) is one of the most widely used strategies in urban planning to reduce traffic injuries (17, 18). A significant reduction of 8–49% in all types of accidents has been reported in sites with FSCs (19, 20). Moreover, fatal and severe injury crashes were reduced by 11–44% in the vicinity of FSC sites (20). However, several studies indicated the sub-optimal efficacy of FSCs on adherence to speed limits in Asian cities (21–23). One of the potential reasons for FSCs' failure to achieve expected effectiveness is a phenomenon called distance halo or kangaroo driving (KD) (23–25). Whether through experience or navigation applications, drivers are informed about the location of FSCs and slow down temporarily while approaching them. According to the literature, the average speed drops by 1.4–18 km/h upstream and recovers to its original level or even higher downstream of FSCs (23, 26, 27). Solutions such as the point-to-point cameras, which average the speed between two points, have helped to solve this problem to some extent. Nevertheless, segments with heavy traffic in point-to-point cameras provide an opportunity to commit speeding in the subsequent segments with light traffic.

An in-depth insight into the factors influencing speeding behavior enables policymakers to adopt optimal preventive strategies in urban planning and design. It is necessary to investigate contributing factors to speeding among taxi drivers, as a population at risk for unsafe driving, in a country with a considerable burden of transport injuries. Therefore, this study aimed to evaluate the influential factors on KD around FSCs among Iranian taxi drivers. We also hypothesized that clustering the FSCs can effectively control speeding behaviors.

## Materials and methods

### Data acquisition

The driving behavior data were collected using Telematics devices that were installed on taxis in the leading three busy terminals of Fars province, Iran, during the 5 months from September 2021. All potential participants were provided with a flier that described the purpose of the study, and drivers who gave written consent were included in the study. Telematics devices used in this study collected the Global Positioning System (GPS) locations of the vehicles along with speed and 3-axis acceleration every 10 s (28). The collected data were sent to the data center *via* the Global System for Mobile Communications (GSM) module embedded inside the devices. Locations of the FSCs were obtained from Road Maintenance and Transportation Organization. Totally, 20 cameras were used for each of the urban and intercity roads. The detection range of cameras was about 20 m for both urban and intercity roads. Among urban cameras, 12 of them were clustered at a regular distance of almost 3.5 km on the Rahmat highway (29). Other cameras were placed either solitarily or at an irregular distance greater than 3.5 km.

The units of analysis in this study were trips per camera, including 2.5 km before and after each camera. For each trip, daylight and weather conditions were included to assess the potential effects of environmental factors on KD. The weather conditions were retrieved from Meteostat (30) and categorized as clear, foggy, rainy, and snowy. Each date's sunrise and sunset times were computed based on the sun's altitude in the Fars province. In addition, to assess KD at different hours, days were divided into six 4-h periods, starting from midnight. In addition, the official calendar of Iran was used to evaluate the difference in KD between holidays and workdays.

### Statistical analysis

Simple linear regression analysis was used to determine the KD trips. Each trip was divided into two segments, before and after the camera, and two simple linear regression models were fitted for each segment as below:


$$\begin{array}{l} Vehicle\;Speed=\text{Average Speed }+\;\left(\text{Time }\times \text{ Acceleration}\right) \end{array}$$

A KD was defined as a trip in which the observed acceleration was significantly higher after the camera as compared to before the camera. To evaluate the effect of the investigated factors on KD, the logistic regression model was utilized. In all statistical analyses, the 0.05 alpha level was used for significance inference and computation of confidence interval (CI).

Apache Spark, version 3.0, a unified big data processing engine, was utilized for data preprocessing (31). All statistical analyses were performed using Statsmodels, version 0.13, an open-source Python library (32).

### Ethical considerations

The study was conducted according to the guidelines of the Declaration of Helsinki and approved by the Research Ethics Committees of the National Institute for Medical Research Development (IR.NIMAD.REC.1399.032, 2019-12-07). All participants signed a written informed consent form allowing their driving data to be used anonymously and confidentially in research projects.

## Results

The implemented telematics infrastructure captured 93,160 trips from 13,857,443 data points of 214 taxis during the study period. All drivers were men, and there was a significant inverse association between drivers' age and KD, with an odds ratio (OR) of 0.98 (95% confidence interval [CI]: 0.98–0.98; *p*-value < 0.01). Totally, 50,742 (54.5%) and 42,418 (45.5%) trips occurred during the daylight hours and nighttime, respectively (Table 1). In addition, 33,484 (35.9%) trips were urban and 59,676 (64.1%) were intercity, 96.9% of which occurred during clear weather. Overall, 2,691 (8.0%) and 17,995 (30.2%) urban and intercity trips were classified as KD, respectively. The intercity trips had significantly higher KD as compared to urban trips with an OR of 4.94 (4.73–5.16; *p*-value < 0.01). However, there was substantial heterogeneity among cameras, with a probability up to 16.67% for urban and 53.27% for intercity trips.

**Table 1:** Distribution of urban and intercity trips, by daylight, time of day, holiday, weather condition, and kangaroo driving (KD).

	**Urban (%)**	**Intercity (%)**	**Total (%)**
**Daylight**			
Daylight	18316 (54.7)	32426 (54.3)	50742 (54.5)
Nighttime	15168 (45.3)	27250 (45.7)	42418 (45.5)
**Time of Day**			
12 A.M.−4 A.M.	2874 (8.6)	4701 (7.9)	7575 (8.1)
4 A.M.−8 A.M.	325 (1.0)	1127 (1.9)	1452 (1.6)
8 A.M.−12 P.M.	7539 (22.5)	14410 (24.1)	21949 (23.6)
12 P.M.−4 P.M.	9752 (29.1)	15501 (26.0)	25253 (27.1)
4 P.M.−8 P.M.	7909 (23.6)	14834 (24.9)	22743 (24.4)
8 P.M.−12 A.M.	5085 (15.2)	9103 (15.3)	14188 (15.2)
**Holiday**			
Holiday	3560 (10.6)	5751 (9.6)	9311 (10.0)
Workday	29924 (89.4)	53925 (90.4)	83849 (90.0)
**Weather Condition**			
Clear	32395 (96.7)	57880 (97.0)	90275 (96.9)
Foggy	640 (1.9)	1119 (1.9)	1759 (1.9)
Rainy	449 (1.3)	677 (1.1)	1126 (1.2)
**Kangaroo Driving (KD)**			
Non-KD	30793 (92.0)	41681 (69.8)	72474 (77.8)
KD	2691 (8.0)	17995 (30.2)	20686 (22.2)

### Daylight

Of the 33,484 urban trips, 18,316 (54.7%) were captured during the daylight hours and 15,168 (45.3%) were recorded at nighttime. There was a statistically significant difference between daylight hours and nighttime in KD for urban trips with an OR of 1.15 (1.06–1.25; *p*-value < 0.01). Among the intercity trips, 32,426 (54.3%) and 27,250 (45.7%) occurred during daylight hours and nighttime, respectively. Similar to urban trips, there was a significant increase in the tendency of drivers toward KD during the daylight hours vs. nighttime on intercity roads (OR: 1.18 [1.14–1.22]; *p*-value < 0.01).

### Time of day

**Figure 1:**
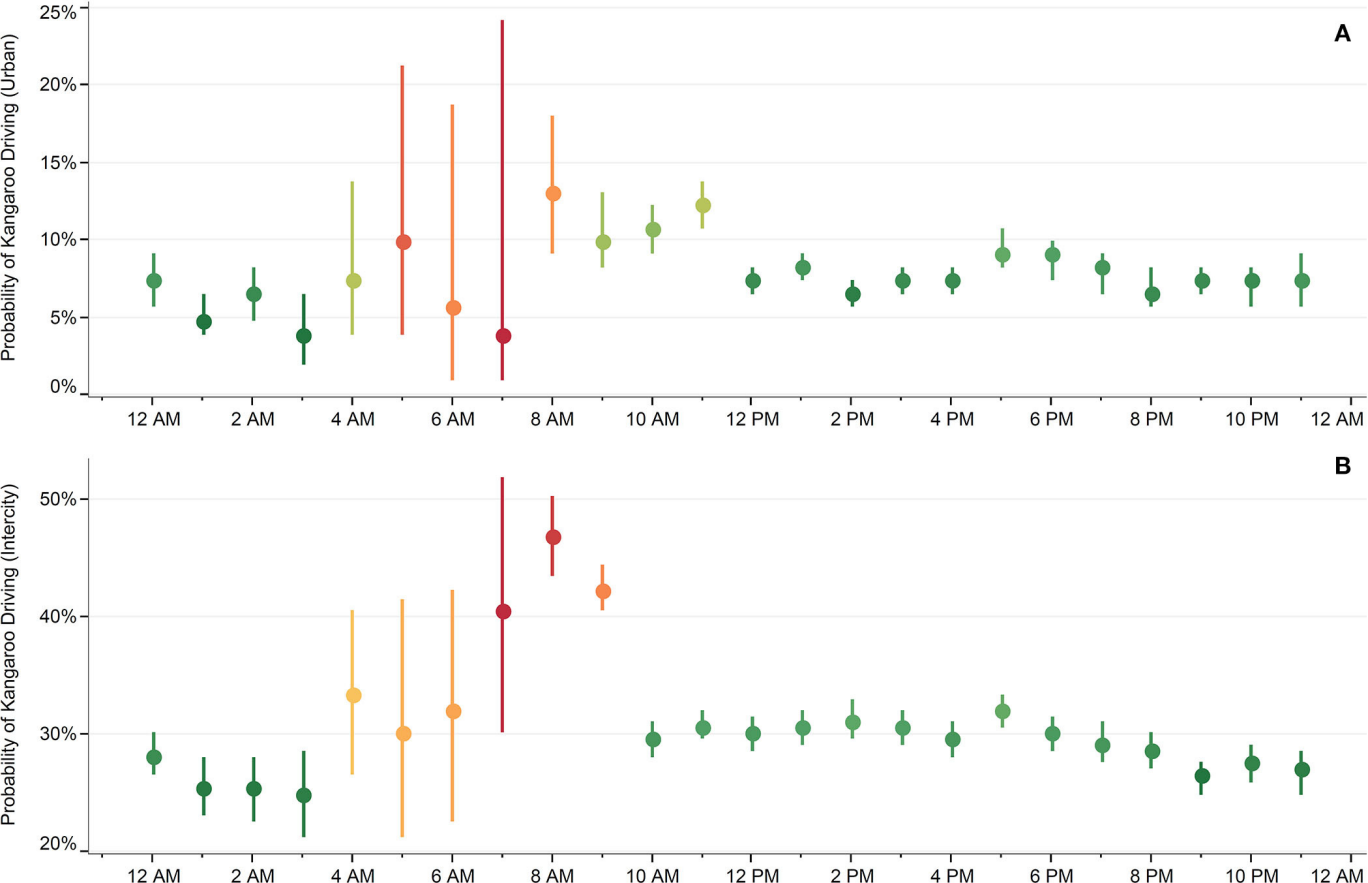
Probability of kangaroo driving (KD) for urban and intercity trips, by the hour of the day. Probability of KD for urban roads **(A)** and intercity **(B)** trips.

The highest number of trips were occurred between 12 and 4 p.m. (25,253 [27.1%]), followed by the 4–8 p.m. (22,743 [24.4%]) timespan. Among different periods, the 4–8 a.m. had the highest probability of KD in both urban (10.71% [7.41–14.53]) and intercity (44.13% [41.18–47.09]) trips. On the contrary, the 12–4 a.m. period with 5.66% (4.76–6.54) and 27.01% (25.37–28.06) for urban and intercity trips, respectively, had the lowest probabilities of KD (Table 2 and Figure 1). The ORs of KD were significantly higher during the 4–8 a.m. as compared to 12–4 a.m. for both urban (1.88 [1.28–2.77; *p*-value < 0.01]) and intercity (2.16 [1.89–2.47; *p*-value < 0.01]) trips.

**Table 2:** Probability and odds ratio (OR) of kangaroo driving (KD) for urban and intercity trips, by the 4-h period of the day.

**Time of Day**	**Urban Odds**	**Urban OR**	**Intercity Odds**	**Intercity OR**
12 A.M.−4 A.M.	5.66 (4.76–6.54)	Reference	27.01 (25.37–28.06)	Reference
4 A.M.−8 A.M.	10.71 (7.41–14.53)	1.88 (1.28–2.77)	44.13 (41.18–47.09)	2.16 (1.89–2.47)
8 A.M.−12 P.M.	9.91 (9.09–10.71)	1.79 (1.51–2.13)	31.97 (31.51–32.89)	1.30 (1.20–1.39)
12 P.M.−4 P.M.	7.41 (6.54–8.26)	1.27 (1.07–1.51)	30.56 (29.58–31.03)	1.19 (1.11–1.29)
4 P.M.−8 P.M.	8.26 (7.41–9.09)	1.44 (1.21–1.72)	30.07 (29.08–30.56)	1.17 (1.09–1.26)
8 P.M.−12 A.M.	7.41 (6.54–8.26)	1.27 (1.05–1.53)	27.01 (25.93–27.54)	1.00 (0.92–1.08)

### Holiday

Among the urban trips, 3,560 (10.6%) and 29,924 (89.4%) were during the holidays and workdays, respectively. The probability of committing KD was significantly lower on holidays, with an OR of 0.87 (0.77–1.00; *p*-value = 0.0498). A total of 5,751 (9.6%) trips were during the holidays and 53,925 (90.4%) were recorded on workdays. In contrast to urban trips, there was no significant difference between the probability of KD on holidays (30.07% [29.08–31.51]) and workdays (30.07% [29.58–30.56]) for intercity trips, with an OR of 1.00 (0.94–1.06; *p*-value = 0.97).

### Clustering

**Figure 2:**
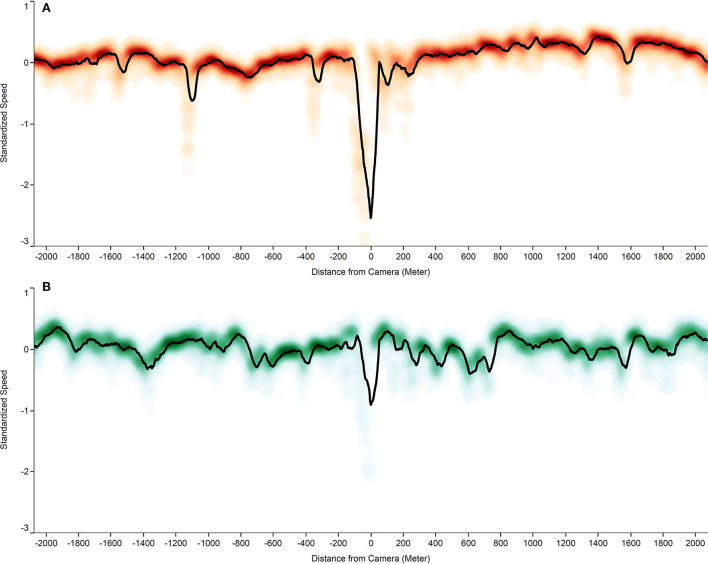
The speed density of vehicles around urban fixed speed cameras (FSCs), by clustering status. **(A)** Standardized speed density of vehicles in the vicinity of solitarily located cameras; **(B)** Standardized speed density of vehicles in clustered camera zone.

Overall, 13,461 (40.2%) of the urban trips took place in the zone of the clustered cameras and 20,023 (59.8%) occurred outside the zone. The probability of committing KD inside and outside the clustered cameras zone was 2.91% (2.90–2.92) and 11.50% (10.71–12.28%), respectively. With an OR of 0.22 (0.20–0.25; *p*-value < 0.01), there was a significant decrease in the odds of KD inside the clustered camera zones. The speed density of vehicles around both non-clustered and clustered urban cameras is shown in Figure 2.

## Discussion

This study tried to discover some of the contributing factors to the speeding behavior around FSCs. The main findings of this study were the significantly lower age of drivers committing KD, the higher KD during daylight hours, the lower KD on holidays on urban roads, and the most remarkable finding of lower KD in clustered camera zones as compared to solitarily placed cameras. In addition, there was substantial heterogeneity in KD among hours of the day, with 4–8 a.m. having almost two times the odds of 12–4 a.m.

Comparing the findings of this study with available literature is not easy since the evidence investigating similar notions is scarce, especially from developing low- and middle-income countries (LMICs). However, KD as a risky driving behavior that leads to higher RTIs could be compared with previous studies on adverse behaviors in driving (24). A meta-analysis of studies investigating the effects of speed cameras on road crashes found the KD behavior in examined populations (33). Moreover, a similar quasi-experimental study on the effects of the FSCs on motorways at two different locations in Belgium also detected significant speed drops upstream of the speed cameras and proposed that this KD behavior could negatively affect driving safety (23). Bringing all these pieces of evidence together suggests more in-depth inquiries on the effect of KD in RTIs and crashes to reach the final statement.

The approaches and solutions to control the KD behavior return to a general rule that is well known as the average speed enforcement in road safety evidence. Literature in this area shows that average speed enforcement has numerous road safety benefits, including improvement of compliance with speed limits, reduction in excessive speeding behaviors, reduction in crash rates, especially those accompanied by fatal outcomes and serious injuries, and improvement of overall traffic flow (34). The mechanism behind the impact of speed enforcement seems to be related to the complex issue of driving behavior and the effect of this enforcement on drivers' attitudes (35). The continuous frequency and patterns of speed enforcement have shown to be more effective in the improvement of speeding behavior (36). The results of a successful experience with the speed enforcement approach in Italy named “Safety Tutor” showed the impact of this program on the reduction of total RTIs and fatal accidents (37). One effective type of average speed enforcement to solve the KD behavior is the automated section speed enforcement system, which measures the average vehicle speed over a long journey instead of a one-point measurement and showed to have significant safety effects and control of driving speed (38). Moreover, the efficacy of this new automated system has been proved in reducing RTIs in high-volume traffic situations and roads (39). The provided evidence mixed with available infrastructure in Iran could improve the use of these tools in enhancing driving behaviors and high-speed issues.

Similar studies previously have investigated the influential roadside factors of speeding behavior. An example of these tools is traffic calming measures, which are traffic lights turning red in case of exceeding speed limits by drivers and are used mostly in rural or intercity roads rather than in urban areas to reduce the risk of RTIs in areas with lower existence of traffic lights (40). It has been shown that the appropriate placement of these tools could prevent excess driving speeds and improve pedestrians' safety passing the roads (40, 41). Considering the importance of pedestrian safety strategies and the pedestrian fatality risk in high-speed driving roads (42), borrowing the experience of these studies could help in controlling the KD challenge.

One of the main findings of this study was the higher efficacy of speed cameras in preventing KD behavior when they are clustered in a section of the road. In the clustered zone, the cameras are about 3.5 km apart, which means that somebody driving at 90 km/h will encounter a camera about every 2 min. The lower occurrence of KD in the clustered cameras zones could be explained by that committing KD behavior multiple times in a relatively short period is potentially tedious and cognitively demanding. In this regard, increasing the number of speed cameras in accident-prone segments of the roads forces drivers to drive in a safer speed range. A previous comparative analysis of road safety among provinces of Iran over 10 years showed that a 10% increase in the number of speed cameras on each 100 km road in rural regions could reduce the RTI fatality rates by merely 0.04% (43). Moreover, FSCs are among the most cost-effective strategies in social policies. It has been reported that increasing the number of speed cameras can significantly reduce medical costs and increase quality-adjusted life years (44). These findings suggest that combining the two strategies of increasing the number of cameras and placing them in accident-prone segments in a clustered fashion can prevent speeding more efficiently than either strategy alone.

This study found a significantly lower mean age of drivers committing KD. The literature has reported age differences in risky driving behaviors and the more substantial impact of positive affect and risk perception in younger drivers (45). Moreover, driving simulation research suggests the considerable contribution of different age groups to risky driving behaviors, mediated by different perceptual and cognitive pathways in diverse age groups (46). In this regard, younger drivers reported more aggressive driving behavior both in focused analysis and considering multiple other variables in the examination (47). The robust part of personality aspects in risky driving behaviors in youth highlights the importance of taking this issue into action by targeted policies (48).

The investigated idea in this study and the patterns of influential factors on the FCSs' effectiveness could beneficially contribute to the development of measures effectively improving road safety and driving behaviors, which are known in the literature as surrogate safety measures (49). Generally, surrogate measures are developed indicators based on various safety analyses and models, helping to reach a more profound knowledge of factors affecting the safety of driving as the focus of this study and leading to the ultimate goal of reducing accidents and road crashes (50). Data driven by safety modeling successfully help to develop and implement such surrogate safety measures to prevent RTIs as much as possible (51). In this regard, numerous dynamic models, such as the Bayesian dynamic value model and static models, could be utilized on the path of developing safety surrogates (52). The results of the current study shed light on the factors and variables that could be incorporated into such models and effectively help scientists in the field of road safety in future studies.

While inspecting the influential factors of road safety and driving behaviors, the role of urbanization and rapid motorization of societies is unavoidable, specifically in developing areas and countries, such as Iran, as the source of the current study (53). Rapid and unplanned urbanization could disproportionately affect the environment, and in the greater image, it could deteriorate different aspects of public health (54). One of these impacts is poor urban and road safety happening in the course of rapid and unsustainable urbanization (53, 55). For example, high traffic density and congestion resulting from such urbanization affect driving safety and behaviors (56). A previous study showed that economic stress and inappropriate urbanization could lead to anger and dangerous driving behaviors resulting in poorer safety on roads (57). Moreover, recent evidence from Iran found that poor urban development might cause aggressive and risky driving behaviors and degrade road safety (58). Putting the evidence together urges the urban and public health authorities toward planning a healthy living environment by focusing on more in-depth factors like what is investigated.

As LMICs bear a heavy burden of RTIs, it is critical to develop and implement road safety plans rigorously. The speed management strategy for the prevention of RTIs was one of the core actions employed in Iran with the help of WHO to achieve a safer road and improve driving behaviors (59). Although there were achievements in this public health issue, subnational assessments showed significant disparities among provinces of Iran in improving safe driving in the past two decades. This notion highlights the need for broader and equitable strategies to reduce RTIs efficiently across the countries (43). Different organizations being responsible for road traffic safety without the proper link between authorities and data governance is a major obstacle to the successful improvement of road safety (60). A thorough investigation of barriers and challenges in preventing RTIs in Iran revealed human factors as the primary contributor; thus, refining behaviors through public education and severer legislation and regulations would be the key to resolving barriers to reach safer driving behaviors (61). Finally, developing a uniform national road safety strategy plan that considers all aspects of road safety and driving behaviors is vital for endorsing all road safety goals and mission activities to reduce RTIs and bring the burden under control (62).

### Strengths and limitations

The most prominent strength of the present study was the utilization of the intricate telematics infrastructure on a large scale, which provides a reliable data source for the assessment of speeding behaviors. The main limitation of this study was the discrete flow of data from telematics devices; however, the 10-s interval of data retrieval provided a near real-time insight into driving behaviors. Nonetheless, the higher resolution of data in future investigations can potentially help to capture steeper cases of KD. Moreover, according to studies, overspeeding is more common among taxi drivers; thus, the study population might be at higher risk for KD. However, this was a limitation of this study only recruiting taxi drivers who also are dominantly male in Iran, which makes the generalization of this study sample to all country population difficult. Nevertheless, the provided in-depth insight on the effectiveness of speed cameras helps implement enhanced speed control strategies, the benefits of which outweigh mentioned limitations.

## Conclusion

Altogether, the findings of this study, specifically the discovered aspects of KD and the contributing factors, could be implemented in further planning to improve safe driving behaviors. The study presented the impact of various factors, such as age, driving hours, driving on holidays, and the major impact of clustered cameras on KD behavior. The heterogeneous occurrence of speeding in different locations and times indicates the necessity of evidence-based decision-making by policymakers. The lower occurrence of KD in clustered camera zones could be a practical key to the effective control of speeding behaviors by helping in the efficient placement of cameras through sustainable development of cities and roads.

## Data availability statement

The data presented in this study are readily available on request from the corresponding author. Restrictions apply to the availability of cameras' location data considering legal issues. Camera locations were obtained from the Road Maintenance and Transportation Organization and are available from the authors only with the permission of Road Maintenance and Transportation Organization.

## Author contributions

Conceptualization: FF and NeR. Methodology: EG and M-RM. Software: M-RM. Validation: M-RM, S-HG, MA-K, and EG. Formal analysis: M-RM and EG. Investigation: SH and M-RM. Resources: NeR, SH, and KL. Data curation: M-RM, S-HG, MM, and MA-K. Writing—original draft preparation: M-RM, SA, and SR-K. Writing—review & editing: FF, KB, NeR, S-HG, MA-K, M-RM, and SS. Visualization: SR-K and M-RM. Supervision: FF, NeR, KL, and NaR. Project administration: NeR and SH. Funding acquisition: NeR.

## Funding

This research was funded by the National Institute of Medical Research Development (NIMAD), Grant No. 983353. The funder had no role in study design, data collection and analysis, decision to publish, or preparation of the manuscript.

## Conflicts of interest

The authors declare that the research was conducted in the absence of any commercial or financial relationships that could be construed as a potential conflict of interest.

## Publisher's note

All claims expressed in this article are solely those of the authors and do not necessarily represent those of their affiliated organizations, or those of the publisher, the editors and the reviewers. Any product that may be evaluated in this article, or claim that may be made by its manufacturer, is not guaranteed or endorsed by the publisher.

## Abbreviations

RTIs: Road Traffic Injuries
FSCs: Fixed Speed Cameras
KD: Kangaroo Driving.
